# Misunderstanding of dosing regimen instructions among patients with chronic diseases receiving polypharmacy at the University of Gondar comprehensive specialized hospital

**DOI:** 10.1371/journal.pone.0280204

**Published:** 2023-01-12

**Authors:** Eyayaw Ashete Belachew, Ashenafi Kibret Sendekie, Samuel Agegnew Wondm, Emneteab Mesfin Ayele, Adeladlew Kassie Netere

**Affiliations:** 1 Department of Clinical Pharmacy, School of Pharmacy, College of Medicine and Health Sciences, University of Gondar, Gondar, Ethiopia; 2 Clinical Pharmacy Unit, Department of Pharmacy, College of Health Sciences, Debre Markos University, Debre Markos, Ethiopia; University of Science and Technology of Fujairah, YEMEN

## Abstract

**Background:**

Misunderstanding dosage instructions in terms of dose, frequency, duration, or any other instruction with patients on polypharmacy is a common problem that leads to the irrational use of medications. This study aimed to assess the level of misunderstanding of dosing instructions among patients with chronic diseases receiving polypharmacy at the chronic outpatient pharmacy of the University of Gondar Compressive Specialized Hospital (UoGCSH).

**Methods:**

An institutional-based cross-sectional survey was conducted from September to November 2021 at the UoGCSH chronic outpatient pharmacy in Northwest Ethiopia. Study subjects were selected by a systematic random sampling method. Drug-drug and drug-food interactions were also checked by Medscape and drug.com to evaluate the significance of interactions. Frequency, percentage, and mean standard deviation (SD) were used to describe the distributions of variables. With a statistical significance level of p < 0.05, the Chi-square test was used to assess the association of variables with the primary outcome.

**Results:**

From a total of 400 participants, more than half (59%) were females, with a mean (SD) age of 57 (±16.3) years old. The study revealed that almost half (50.8%) of the participants misunderstood at least one dosage instruction, and around two-fifths (38.5%) misunderstood the frequency of drug administration. More than 90% of patients had no understanding of drug-drug interactions (DDIs). Sex (X^2^ = 16.837; P<0.0001), educational level (X^2^ = 50.251; P < 0.0001), residence (X^2^ = 5.164; P < 0.023) and duration of stay on medication (X^2^ = 13.806; P < 0.0003) were significantly associated with misunderstanding of dosage regimen instructions.

**Conclusion:**

The study showed that a significant number of patients did not understand their medication dosage regimen instructions. To address these significant drug-related issues, healthcare providers could effectively engage in interventions such as written instructions accompanying patients and additional counseling.

## Introduction

Appropriate use of medicines has been promoted as the cornerstone of medication therapy, focused primarily on ensuring rational prescription habits and dispensing quality, though patients’ knowledge of dispensed medicine has been overlooked. It is agreed that the name and purposes of the use of medication, the dose, dosage frequency, and treatment duration should be included in the knowledge of medication use by the patients [1–3].

Patient compliance is crucial to achieving the best possible drug treatment outcome [4]. However, patient misunderstanding of dosage regimen instructions is common and a potential root cause for medication error, poor medication adherence, and subsequent unintentional misuse of a prescription drug, which is a cause for worse health outcomes [1, 5, 6]. The duration of treatment and the dosage regimen, as well as the time of drug administration, are both regarded as crucial aspects of drug therapy [7]. Medication administration is a common problem for patients, particularly in outpatient settings because of the lack of medication sources compared to inpatient settings [8]. It is estimated that a large number of outpatient medication errors occur as a result of patient noncompliance, which could be due to medication looks and sounds similar [1].

Patients expect to receive the necessary information from pharmacists to support quality pharmaceutical use through counseling, which could be accompanied by verbal and written instructions as well as labeling attached to the immediate container of administered medicines, which is the only tangible source of dispensed medication [9]. However, this is frequently inconsistent, fragmentary, and difficult to read and comprehend for the patients [10], which leads to incorrect interpretation of medication usage. Incorrect interpretation of drug usage can result in medication mistakes and/or adverse events [8]. A study in the United States (US) has shown that one-third of the 1.5 million adverse events occurred in the outpatient context [11, 12], and a study conducted in Singapore found that polypharmacy is linked to inappropriate medication use and increased hospitalization [13–15].

Though pharmacists play an important role in informing patients about drug-related information like duration of therapy, common side effects, therapeutic indications, and contraindications [16], a recent study conducted at Dessie Referral Hospital in Northeast Ethiopia revealed that about 77.34% of the patients had misunderstood one or more dosage instructions, which increases medication non-adherence Lacking drug adherence is caused by a failure to offer relevant information in an easy and understandable manner, which can be remedied by patient counseling and education. The lack of medication dosage instruction and misunderstanding might be due to patients’ behaviors and pill or injection burdens, all of which may cause poor medication adherence, particularly in patients with a higher number of medications [17]. However, in Ethiopia, counseling services are provided roughly 60% of the time, which is below the standard [18, 19]. Because medication errors can take many forms, a misinterpretation of the instructions on prescription container labels, which frequently leads to erroneous medication administration, is among the most common medication errors [20]. As a result, Ethiopian pharmacists are expected to provide clear and comprehensive information on drugs, which receives little attention in Ethiopia. Patients’ inability to understand instructions due to a lack of oral and written information from doctors and/or pharmacists on how to manage prescription medication has resulted in therapeutic failure. According to studies, 46% of patients misread one or more dosage the recommendations [7] and one or more auxiliary warnings [8].

Because there is limited literature in the study area, this study is expected to provide up-to-date information on chronic patients’ misunderstanding of dosage instructions and the extent of polypharmacy. Implementing the recommendations derived from the study’s findings will allow us to reduce the problems caused by misunderstanding of dosage instructions and improve treatment outcomes. According to the best of the authors’ searches, this is the initial study on the misunderstanding of dosage instructions among patients with chronic diseases on polypharmacy in the study area. As a result, this study aimed to figure out the depth of the problem related to misunderstanding medication dosage regimen instructions. Therefore, the aim of study was to assess the misunderstanding of dosing instructions among patients with chronic diseases on polypharmacy attending the chronic outpatient pharmacy at the University of Gondar comprehensive and specialized hospital (UoGCSH).

## Methods and materials

### Study design and setting

An Institutional-based cross-sectional study was conducted at the UoGCSH chronic outpatient pharmacy unit from September to November 2021. The hospital is located in Gondar, Northwest Ethiopia, which is 738 km away from the capital city of Ethiopia, Addis Ababa. The hospital has seven pharmacy units, in which pharmacy professionals provide service to patients, including emergency pharmacy, chronic outpatient pharmacy (OPD1), ambulatory outpatient pharmacy (OPD2), inpatient pharmacy (IPD), gynecology and obstetrics pharmacy, optometry pharmacy, and antiretroviral treatment pharmacy (ART).

### Study participants and inclusion criteria

Chronic patients receiving polypharmacy, whose age is 18 years and above, who attended the UoGCSH chronic outpatient pharmacy for follow-up were eligible for this study. Chronic polypharmacy patients who had received their medications during the study period were also included in the study. However, patients who did not volunteer to participate in the study, seriously ill patients unable to communicate, patients with serious psychiatric or cognitive impairment, and those patients administered their medications by health professionals were excluded from the study.

### Sample size determination and sampling procedure

The size was determined by using the single population proportion formula: n = Z^2^pq/d^2^, considering a 50% (P = 0.5) proportion of patients with misunderstanding of dosage instructions of medication because there was no literature in the study area regarding the study topic; n = the minimum sample size (Z = 1.96) at a 95% confidence interval (d = 0.05) with a tolerated margin of sampling of 5. Finally, by adding a 5% contingency of non-respondents, a total of 405 patients were approached in the study.

The participants were included in the study using a systematic random sampling method. According to the chronic pharmacy records of the University of Gondar, on average, 700 chronic patients with polypharmacy visited the ambulatory care center per month (keeping in mind that all chronic patients will be attended to for one to three months at the UoGCSH). Chronic patients are advised to visit the ambulatory care for a minimum of one month and a maximum of three months. As a result, adding all of the patients’ average three-month visits would result in approximately 2100 chronic patients with polypharmacy who were visited every three months. Taking into consideration that the sample was collected within three months, this makes the sampling fraction (k-interval) 2100/405 = 5. The initial study subjects were chosen at random, and then every five people, study individuals were chosen at random, and their relevant data was collected. Whenever the patient on hand was not eligible, the next immediate one was selected, and the same approach was followed throughout the entire data collection procedure.

### Data collection instruments, procedures and quality management

The data collection format was initially prepared in English. It was then translated to the local language (Amharic) and back-translated to English to maintain consistency. Trained pharmacy professionals, under the investigator’s daily supervision, collected the data. During data collection, face-to-face interviews and direct observation of dispensed medications were used, with a structured questionnaire for interviews consisting of both closed and open-ended questions addressing socio-demographic variables and variables addressing patients’ understanding of dosage regimen instructions [1, 5]. Drug-drug interaction checker templates were prepared for commonly used drugs in order to stress-free the data collection. The face validity of the survey was assessed among three clinical pharmacy expert personnel for the clarity of the questions. Then, the questionnaire was pre-tested for content, design, readability, and comprehension by 20 people. Based on the results obtained, the questionnaire was modified. The Cronbach alpha was done to measure the internal consistency of the questionnaire and it was (α = 0.898) and modifications were made based on the response. As a result, the survey was simple to understand and answer. Furthermore, the investigators had provided feedback and corrections on a daily basis to the data collectors. Completion, accuracy, and clarity of the collected data were checked carefully on a regular basis. The questionnaires had been randomly selected for quality control purpose and rechecked by an experienced pharmacist. In addition, the study participants were clearly informed about the purpose of the survey, and thereby creating a friendly atmosphere.

### Data entry and analysis

The collected data was cleared, categorized, and analyzed using Statistical Package for Social Sciences (SPSS) version 26, and the results were presented in tables and figures as necessary. With a significance level p of 0.05, the chi-square test was used to look for any association between outcome variables and independent variables.

### Operational definition

#### Polypharmacy

In this study, polypharmacy indicates the prescription to a patient of three or more drugs [21, 22]

#### Misunderstanding

Indicates whether patients with chronic illnesses who are on polypharmacy fail to comply with the dose, duration, frequency, or any other instructions [1, 6].

A food-drug interaction: it is a situation in which the drug interacts with food, beverages, or dietary supplements that the person is consuming [23].

A drug-drug interaction occurs a situation when one drug interacts with another that the person is taking [23].

### Ethical consideration and confidentiality

The study was approved by the ethics approval committee of the University of Gondar with reference number of SOP/262/2021. Then, letter of permission was obtained from chronic pharmacy of UoGCSH. Both Verbal and written informed consent was obtained from each study subject prior to the interview after the purpose of the study is explained to them. Confidentiality of the information was assured.

## Results

The final study included 400 patients (a response rate of 99.3%) out of a total of 403 approached. More than half (59%) of the participants were females, with a mean (SD) age of 57 (±16.3) years old. From the total respondents, 353(88.3%) were Amhara in ethnicity, followed by Tigre, and the majority (65.5%) were residing in urban areas. Regarding the respondent’s religion, most of the participants (85.5%) were orthodox Christians. Approximately 29.5% of the participants were unable to read or write (**Table 1**).

**Table 1:** Sociodemographic characteristics among patients with chronic disease at UoGCSH, 2021(N = 400).

Socio-demographic characteristics Variables	Categories	Frequency (%)
Age	15–24	18(4.5)
	25–34	24(6.0)
	35–44	48(12.0)
	45–54	100(25.0)
	55–64	105(26.3)
	≥65	105(26.3)
Sex	Male	164 (41.0)
	Female	236(59.0)
Religion	Orthodox	342(85.5)
	Muslim	54(13.5)
	Protestant	4(1.0)
Ethnicity	Amhara	353(88.3)
	Tigre	47(11.8)
Educational status	Cannot read / write	118(29.5)
	Can read / write	133(33.3)
	Grade 1–6	41(10.3)
	Grade 7–12	56(14.0)
	Diploma and above	52(13.0)
Occupation	Governmental employee	35(8.8)
	Self- employee	164(41.0)
	NGO-employee	10(2.5)
	Retired	21(5.3)
	House wife	153(38.3)
	Other	17(4.3)
Marital status	Unmarried	30(7.5)
	Married	345(86.3)
	Other	25(6.3)
Residence	Rural	138(34.5)
	Urban	262(65.5)
Duration of medication use	1–6 month	107(26.8)
	7-12month	38(9.5)
	1-4years	204(51.0)
	5 and more years	51(12.8)

### Rate of misunderstanding of the recommended instructions

Of the total 400 respondents, overall, more than half (50.8%) of the patients incorrectly understood one or more dosage regimen instructions, and the majority of the respondents (93.5%) also incorrectly understood drug-drug interactions. However, the rest of the instructions had more correct understanding than incorrect understanding (**Table 2**).

**Table 2:** Proportion of the study participants with respective understanding level of dosage regimen instructions (N = 400).

Instructions	Correct understanding n (%)	Incorrect understanding n (%)
One or more dosage regimen instruction	197(49.3)	203(50.8)
Amount of dose administered	316 (79)	84(21)
Frequency of dose administration	246 (61.5)	154(38.5)
Duration of treatment	385(96.3)	15(3.8**)**
Food-drug interaction	366(91.5)	34(8.5)
Drug-drug interaction	26 (6.5)	374(93.5)

### Misunderstanding of dosage regimens instructions stratified by Socio-demographic characteristics

From the participants’ socio demographic characteristics, the occupation of respondents was significantly associated with misunderstanding of the dose (X^2^ = 17.823; P = 0.003), misunderstanding of the frequency (X^2^ = 25.537; P = 0.0001), and misunderstanding of the duration of treatment (X^2^ = 12.012; P = 0.035). Similarly, the sex of the participants had significant association with misunderstanding of dose (X^2^ = 16.837; P = 0.0001), and misunderstanding of frequency (X^2^ = 3.971; P = 0.046) and duration of treatment (X^2^ = 4.931; P = 0.026) (**Table 3**).

**Table 3:** Misunderstanding of dosage regimen instructions stratified by socio-demographic characteristics.

Socio-demographic characteristics		Misunderstanding dosage regimen instructions		
		Dose n (%)	Frequency of administration n (%)	Duration of treatment n (%)
Age	15–24	3(3.6)	5(3.2)	1(6.6)
	25–34	6(7.14)	8(5.2)	0(0.0)
	35–44	7(8.3)	12(7.7)	2(13.3)
	45–54	19(22.6)	36(23.2)	4(26.7)
	55–64	24(28.6)	46(29.7)	4(26.7)
	≥65	25(29.8)	48(31)	4(26.7)
	Association	X^2^ = 2.585 P<0.764	X^2^ = 8.630 P<0.125	X^2^ = 1.140 P<0.950
Sex	Male	18(21.4)	54(34.8)	2(13.3)
	Female	66(78.6)	101(65.2)	13(86.7)
		**X**^**2**^ **= 16.837** P<0.0001*	**X**^**2**^ = 3.971 P<0.046*	**X**^**2**^ = 4.931 P<0.026*
Religion	Orthodox	69(82.1)	131(84.5)	14(93.3)
	Muslim	15(17.9)	24(15.5)	1(6.7)
	Protestant	0(0.0)	0(0.0)	0(0.0)
	Association	X^2^ = 2.699 P<0.259	X^2^ = 3.297 P<0.192	X^2^ = 0.807 P<0.668
Ethnicity	Amhara	73(86.9)	132(85.2)	12(80)
	Tigre	11(13.1)	23(14.8)	3(20)
	Association	X^2^ = 0.186 P<0.667	X^2^ = 2.328 P<0.127	X^2^ = 1.023 P<0.312
Educational status	Cannot read / write	32(38.1)	68(43.9)	6(40)
	Can read / write	29(34.5)	59(38.1)	6(40)
	Grade 1–6	6(7.1)	10(6.5)	0(0.0)
	Grade 7–12	14(16.7)	15(9.7)	3(20)
	Diploma and above	3(3.6)	3(2)	0(0.0)
	**Association**	**X**^**2**^ = 11.527 P<0.021*	**X**^**2**^ = 50.251 P<0.0001*	X^2^ = 4.820 P<0.306
Occupation	Governmental employee	6(7.1)	2(1.3)	0(0.0)
	Self- employee	24(28.6)	66(42.6)	3(20)
	NGO-employee	0(0.0)	1(0.62)	0(0.0)
	Retired	4(4.8)	8(5.2)	0(0.0)
	House wife	48(57.1)	73(47.1)	12(80)
	Other	2(2.4)	5(3.2)	0(0.0)
	Association	**X**^**2**^ = 17.823 P<0.003*	**X**^**2**^ = 25.537 P<0.0001*	**X**^**2**^ = 12.012 P<0.035*
Marital status	Unmarried	4(4.8)	7(5)	0(0.0)
	Married	73(87)	132(85.2)	13(86.7)
	Other	7(8.3)	16(10.3)	2(13.3)
	Association	X^2^ = 1.807 P<0.405	X^2^ = 9.755 P<0.008	X^2^ = 2.420 P<0.298
Residence	Rural	31(37)	64(41.3)	7(46.7)
	Urban	53(63.1)	91(58.7)	8(53.3)
	Association	X^2^ = 0.272 P<0.602	**X**^**2**^ = 5.164 P<0.023*	X^2^ = 1.021 P<0.312
Duration of medication use	1–6 months	34(40.5)	47(30.3)	5(33.3)
	7–12 months	10(12)	19(12.3)	3(20)
	1–4 years	35(41.7)	71(46)	6(40)
	5 and more	5(6.0)	18(12)	1(6.7)
	Association	**X**^**2**^ = 13.806 P<0.0003*	X^2^ = 4.829 P<0.185	X^2^ = 2.883 P<0.410

Bold texts show variables with significant association

* indicates P < 0.05.

### Distribution misunderstood medications for dosage regimen instructions

From a total of 1399 dispensed drugs, 282 (20.15%) were misunderstood by study participants. From these, the majority accounted for a misunderstanding of the frequency of drug administration (179, 63.4%), and the least was a misunderstanding of the duration of drug therapy (17 or 6%). The major classes of drugs associated with misunderstanding of dosage regimen instructions were cardiovascular drugs (146, 51.8%) (**Table 4**).

**Table 4:** Distribution of misunderstood medications by study participants for dosage regimen instructions.

Class of drugs	Number of medications misunderstood		
	Dose N (%)	Frequency of administration N (%)	Duration of treatment N (%)
Anti-diabetics	45(11.3)	27(6.8)	0(0.0)
Cardiovascular	22(5.6)	71(17.8)	3(0.8)
Antipsychotic	8(2.1)	10(2.6)	2(0.5)
Dyslipidemia	0(0.0)	1(0.3)	0(0.0)
Antiasthmatic	3(0.8)	18(4.6)	1(0.3)
Antiplatelet	0(0.0)	3(0.8)	0(0.0)
Anticoagulant	7(1.8)	2(0.5)	1(0.3)
Anti-thyroid	0(0.0)	26(6.5)	0(0.0)
Other medication(drugs)	1(0.3)	21(5.3)	10(2.6)

### Number of prescribed medications and prevalence of drug-drug interactions

**Fig 1:**
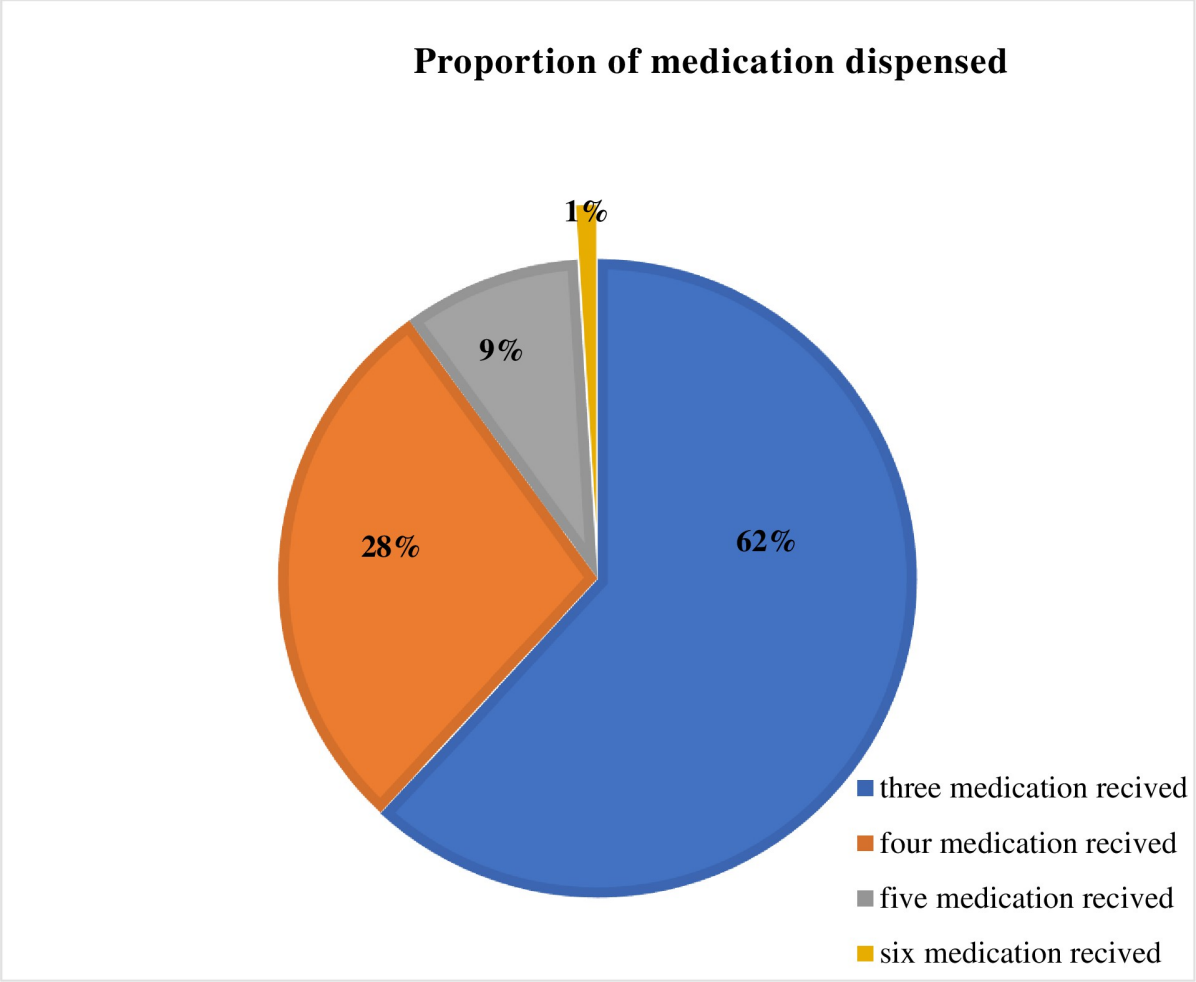
The proportion of participants with the respective number of dispensed medications among patients with chronic disease at UoGCSH, 2021.

Regarding the number of medications received, the majority of the study participants (62%) had received three medications (**Fig 1**).

**Fig 2:**
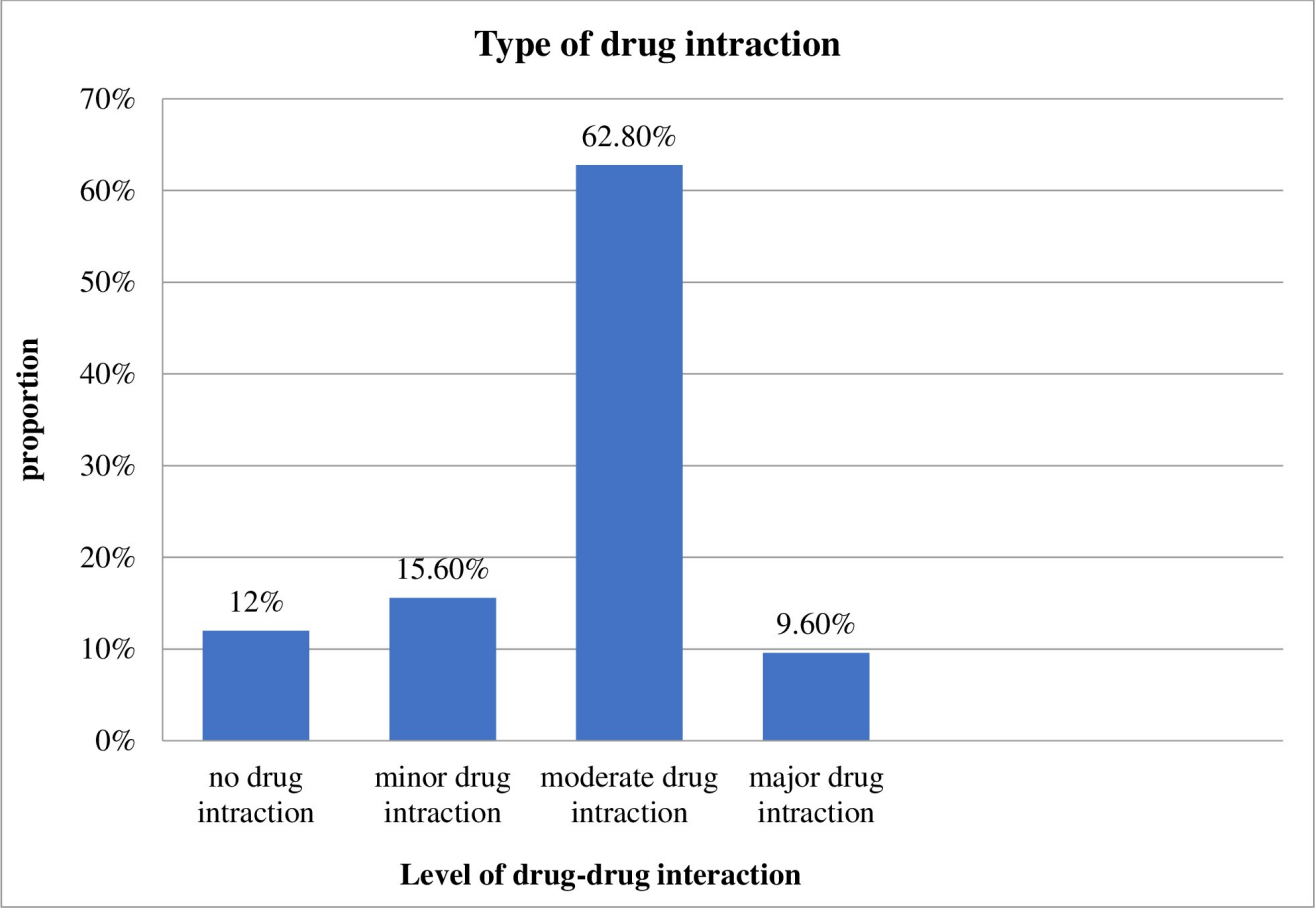
The level of drug-drug interactions in the study participants.

Concerning DDIs, more than half of the respondents (62.8%) were exposed for a moderate level of DDIs (**Fig 2**).

## Discussion

The current study assessed the level of patients’ misunderstanding of dosage regimen instructions for their dispensed medications at the outpatient chronic pharmacy of UoGCSH. Most medication counseling provided by pharmacists is assumed to be understandable by patients. However, in this study, more than half (50.8%) of the participants misunderstood one or more of dosage regimen instructions given by pharmacy professionals. In fact, this is significantly lower than compared to others studies: 72.4% at Nekemte referral hospital [1], 77.3% at Wollega [5], and 77.3% at Dessie [18]. The discrepancy might be due to differences in study population across the study settings. Our study was conducted on chronic outpatient pharmacy among chronic patients receiving polypharmacy. The other studies, on the other hand, were conducted on patients who visited an outpatient ambulatory pharmacy. Hence, chronic pharmacies often treat patients with chronic conditions that require regimented medication protocols; they provide patients with more detailed education and patient care services that are required for their medications. Additionally, patients with chronic diseases have better experience and exposure with their medication regimen instructions, which can help ensure medication adherence and increase their medication regimen instruction understanding. However, ambulatory outpatient pharmacies have been given service for any disease and handle a variety of prescriptions and over-the-counter medications for treating common illnesses. So, most patients who visited ambulatory outpatient pharmacies had no previous knowledge regarding the diseases and medications they used as chronic patients. As a result, the discrepancy in our findings would be because of this. As well as the discrepancy being due to sociodemographic differences like the educational status, attitudes and knowledge of the study populations. The other possible explanation might be due to a difference in the level of communication between the pharmacist and patients because of different barriers like language and others.

On the other hand, the result is higher than a study done in the USA (46.3%) [24] and Brazil (38.2%) [25]. This variation could be attributed to differences in the study countries’ medication counseling policies, pharmacists’ engagement and communication with patients, and the health literacy status of the study population. The other possible explanation might be due to the fact that most patients visiting the outpatient clinic may have a long waiting time for examination and history taking, or laboratory tests, and for diagnostic procedures. Finally, patients may feel tired and become less attentive to medication counseling provided by pharmacy professionals, which can ultimately lead them to misunderstand the instructions given, as a study showed that a lack of attention to the warning labels has been recognized as a problem [24].

In this study, nearly two-fifths (38.5%) of study subjects misunderstood the frequency of drug administration, which is significantly lower than compared with previous studies at Dessie referral hospital (51.3%) [18], Shambu primary hospital, Southwest Ethiopia, 66% [5], and Nekemte, Southwest Ethiopia, 67.6% [1]. This may be due to the fact that the study subjects in the current study were chronic patients may have been familiar with their medications for a long period of time. Similarly, the current figure was lower than the earlier study conducted in the US, which reported that 79% of patients were taking all TID base doses within 12 hours [26]. This may be due to the fact that the earlier study was done only on medications prescribed at the TID frequency of drug administration, whereas the present study included all medications prescribed at different dosing frequencies, such as, PRN, QD, BID, TID, QID, and the like.

Regarding to the level of dosing instruction understanding, around one-fifth (21%) of the study participants incorrectly understood their instruction of daily dose. A significant number of respondents who were unable to read or write misunderstood the dose instructions. It is in line with another study conducted at Nekemte referral hospital [1], except the figure was slightly higher in this study. Another study also disclosed that the majority of patients with low literacy levels who cannot read or write could not demonstrate the number of pills to be taken daily [24, 26]. The finding suggests that patients with low literacy levels need particularly a strict counseling and instructions for their medications. Therefore, pharmacists could have a substantial contribution to make in helping patients understand the appropriate dosing of their medications. A significant proportion of the study participants incorrectly understood their instructions regarding the duration of treatment. However, when compared to other studies [5, 27], a lower proportion of respondents misunderstood the treatment duration instruction in this study. It is justified that the study participants in this study may be a little bit aware of the duration of treatment due to their repeated exposure, which may help them to be more familiar than other patients.

Generally, the finding may imply that such a high extent of patient misunderstanding of dosage regimen instructions clearly alerts the healthcare providers, the hospital administrators, and policy makers to design an effective medication counseling policy and procedures so as to minimize the negative consequence of patient misunderstanding in medication use, which will have a significant impact on the healthcare system. Several reports from both developed and developing countries identified incorrect dispensing and use of incorrect doses as major causes of irrational drug use [1, 2, 18, 25, 28]. Particularly, patients with polypharmacy may have a high burden of misunderstanding and poor adherence to their medications. An earlier study conducted in a similar setting also proved that patients with a higher number of medications had poor medication adherence [17]. Thus, it is the pharmacy professional’s responsibility to provide pharmaceutical care that meets the medication needs of the patient. This care must not only be precise in the manual aspects of filling the prescription order but also provide the patient with necessary information and guidance to ensure that the patient’s compliance in taking the medication is proper. The pharmacist can help the patient avoid medication misuse and latent errors at home by providing them with adequate information on medication safety [5, 27, 29]. Therefore, pharmacy professionals could be vigilant to confirm patients’ understanding through the “teach-me-back” method, in which patients are asked to repeat instructions to demonstrate their understanding, especially those vulnerable groups of the population, like patients with low health literacy [24].

The current study has its own strengths and some limitations. The current study is the first of its kind in Ethiopia to evaluate dosing regimen instruction in patients with chronic disease on polypharmacy, particularly in the study setting. As a result, this study may be useful for prescribers and dispensers in tailoring recommendations and interventions for their patients. It is also critical for policymakers and healthcare administrators to create a new manual for the dispensing and counseling of medications in polypharmacy patients. During the data collection phase, the current study also provided a template for commonly prescribed chronic drugs to check drug-drug interaction and drug-food interaction. As a limitation, the current study did not show an association between the level of misunderstanding of dosage instructions and the patients’ actual medication taking behavior.

## Conclusion

Generally, in this study, a significant proportion of participants incorrectly understood the dosage regimen instructions in terms of dosing, frequency, duration, drug-drug interaction, and drug-food interaction. Patients with low literacy levels, in particular, are prone to misinterpreting dosing instructions. Therefore, the healthcare providers, especially the prescribers and dispensers, could be vigilant to improve their patients’ understanding. The hospitals and health bureau administrators could design an effective medication counseling policy and procedures so as to minimize the negative consequences of patients’ misunderstanding of medication dosage regimens.

## Supporting information

S1 File Mini dataset: A data set used to analyze and generate the data. (SAV)

## Abbreviations

DDI: Drug-drug interactions
UoGCSH: University of Gondar Comprehensive specialized hospital
WHO: World Health Organization
